# Measuring Intensity of End of Life Care: A Systematic Review

**DOI:** 10.1371/journal.pone.0123764

**Published:** 2015-04-14

**Authors:** Xhyljeta Luta, Maud Maessen, Matthias Egger, Andreas E. Stuck, David Goodman, Kerri M. Clough-Gorr

**Affiliations:** 1 Institute of Social and Preventive Medicine (ISPM), University of Bern, Bern, Switzerland; 2 University Department of Geriatrics, Inselspital Bern, Bern, Switzerland; 3 The Dartmouth Institute of Health Policy & Clinical Practice, Lebanon, NH, United States of America; 4 Section of Geriatrics, Boston University Medical Center, Boston, MA, United States of America; Universidade do Extremo Sul Catarinense, BRAZIL

## Abstract

**Background:**

Many studies have measured the intensity of end of life care. However, no summary of the measures used in the field is currently available.

**Objectives:**

To summarise features, characteristics of use and reported validity of measures used for evaluating intensity of end of life care.

**Methods:**

This was a systematic review according to PRISMA guidelines. We performed a comprehensive literature search in Ovid Medline, Embase, The Cochrane Library of Systematic Reviews and reference lists published between 1990-2014. Two reviewers independently screened titles, abstracts, full texts and extracted data. Studies were eligible if they used a measure of end of life care intensity, defined as all quantifiable measures describing the type and intensity of medical care administered during the last year of life.

**Results:**

A total of 58 of 1590 potentially eligible studies met our inclusion criteria and were included. The most commonly reported measures were hospitalizations (n = 44), intensive care unit admissions (n = 39) and chemotherapy use (n = 27). Studies measured intensity of care in different timeframes ranging from 48 hours to 12 months. The majority of studies were conducted in cancer patients (n = 31). Only 4 studies included information on validation of the measures used. None evaluated construct validity, while 3 studies considered criterion and 1 study reported both content and criterion validity.

**Conclusions:**

This review provides a synthesis to aid in choosing intensity of end of life care measures based on their previous use but simultaneously highlights the crucial need for more validation studies and consensus in the field.

## Introduction

As the world’s population ages, research on end of life care is increasingly important. Healthcare expenditures in the last year of life are, on average, five times higher than in other years [1]. Health services near the end of life are often responsible for much of the increased costs since many patients die in acute care settings [1, 2]. Efforts to improve end of life care require accurate measurement of the care provided.

Healthcare costs at the end of life are directly related to the intensity of care. Intensity of end of life care is usually highest in hospital settings. Evidence suggests that in the days just before death patients commonly receive invasive or life prolonging procedures. For example, studies have shown about half of patients at the end of life receive mechanical ventilation, undergo chemotherapy, and a quarter receive cardio pulmonary resuscitation (CPR) [3–6]. Although these practices are common, they do not always align with patients’ preferences.

Nearly 40% of patients die in acute care hospitals [7]. Yet studies report between 45%-86% of patients at the end of life say they would prefer to die at home [7–10]. End of life treatments may be more influenced by factors like local practice patterns and number of hospital beds than by patients’ preference [10–13]. Consideration of costs and clinical outcomes are key to understanding the quality of end of life care, however, appropriateness of care is ultimately judged by patients’ preferences.

Previous studies have highlighted issues that arise when measuring end of life care [14, 15]. Defining the end phase of life can be ambiguous and terms are often used differently between clinical settings, healthcare professionals and researchers. There are many different illness trajectories for dying people, and there is no accurate clinical indicator to predict time of death [16, 17]. As a result definitions for the end of life phase vary considerably. Furthermore, in order to measure intensity of care at the end of life, it is essential to first define what is meant by both intensity and end of life care. Intensity of care attempts to identify high levels of utilization and not to merely quantify healthcare use. End of life care generally considers all health care administered in a distinct timeframe before death. It is often used interchangeably with various other terms such as palliative care, hospice care, or terminal care [18]. This lack of agreement presents methodological challenges when conducting and comparing end of life research [19, 20].

Despite these challenges many studies have measured the intensity of care at the end of life [11, 21–23]. However, researchers disagree on the standards of measurement, and no overview of the measures used in the field is currently available [21]. A plethora of measures have been used in previous research—most commonly high usage of hospitals, the number of physician visits, CPR, mechanical ventilation, tracheostomy, and chemotherapy near the end of life [21, 24, 25].

Studies of the intensity of end of life care require reliable and valid measures that work in different care settings, populations and diseases. However, it is currently unclear what tools have adequate validity and should be recommended for measuring intensity of end of life care. In this systematic review, we provide a comprehensive overview of the measures of intensity of end of life care that are currently used in published original research. We summarize their features (i.e., type of measures) and describe characteristics of their use (i.e., population, timeframe) and reported validity.

## Methods

The methods to identify, select, and critically appraise the relevant studies in this systematic review are reported according to the PRISMA (preferred reporting items for systematic reviews and meta-analyses) guidelines [26].

### Definition

We defined measures of intensity of end of life care as all quantifiable measures describing the type and intensity of medical care administered during the last year of life. We included the following categories of care: hospitalizations (acute hospital, intensive care unit [ICU]; emergency department [ED]); potentially life-sustaining invasive procedures which include a range of treatments administering complex, invasive methods to prolong a person's life (e.g., resuscitation, intubation, and mechanical ventilation, artificial feeding, dialysis); and potentially life-prolonging treatments (e.g., surgery, chemotherapy, radiation, medical imaging, transfusions).

### Literature search and eligibility criteria

Studies were included in this systematic review based on the following criteria: (1) used a measure that met our definition and (2) explicitly stated they were measuring intensity of end of life care. We included cohort studies (prospective and retrospective), case-control studies, and randomised-controlled trials. We included all studies that used the term intensity or a synonym (intensive, aggressive, extensive) or that indicated they were quantifying higher or increased levels (frequencies, rates) of end of life care. We searched for studies reporting measures of intensity of end of life care in adults aged 18 or older. We excluded studies on children and patients with mental illness because these population groups have different care needs and thus may require different measures of intensity of care. We also excluded studies that: (1) did not include a clearly defined end of life timeframe (e.g. measured within 30 days before death); (2) reported exclusively on cost; (3) included exclusively clinical palliative care; (4) evaluated only outpatient settings; (5) were case reports; or (6) were published before 1990.

OVID Medline, EMBASE, and the Cochrane Database for Systematic Reviews were searched to identify studies from 1 January 1990 up to 29 January 2014. We used the following key words: end of life care; last year of life; last months of life; terminal care; terminally ill; critically ill; palliative care; treatment intensity; intensity of care; intensity of treatment; aggressiveness of care; amount of care; health services utilization. A specific search strategy was developed for each database. No language restrictions were applied. We identified additional studies by hand-searching the reference lists of included studies. Appendix 1 provides detailed information on search terms.

Two reviewers (X.L. and M.M.) independently screened titles, abstracts, and full texts. Disagreements about inclusion and exclusion were resolved through consensus with a third reviewer (K.C.).

### Data extraction and quality assessment

We extracted the following information for each study: author; year of publication; country; aim; design; details on the study populations (disease, age, and gender); setting (e.g., emergency department, intensive care unit); and a description of the measure. When studies used more than one measure, or included more than one population (multiple comparison groups), we only extracted data on the measures that met our inclusion criteria.

We developed an individualized assessment checklist that included both validation of individual measures and criteria for quality of methods used. We extracted data on the validity of measures [27, 28], including face and content validity (measure covers the domains considered to be important, assessment is based on the subjective views of experts); criterion validity (measure correlates with another instrument that measures similar aspects, preferably a reference standard or one that is widely used and accepted in the field); and construct validity (measure conforms with the results using other established scales or different groups of patients).

We rated the quality of evidence as: “good” (reported measure validity, measure was used in other studies, well-described study design, thorough assessment of potential sources of bias well documented strengths and limitations); “moderate” (no reported measure validity and met three or more other criteria); and “low” (no reported measure validity and met less than three other criteria). We contacted developers of measures and sought additional information on validity of measures. Two reviewers (X.L. and M.M.) independently extracted data from each included study.

## Results

### Identification of eligible studies

**Fig 1:**
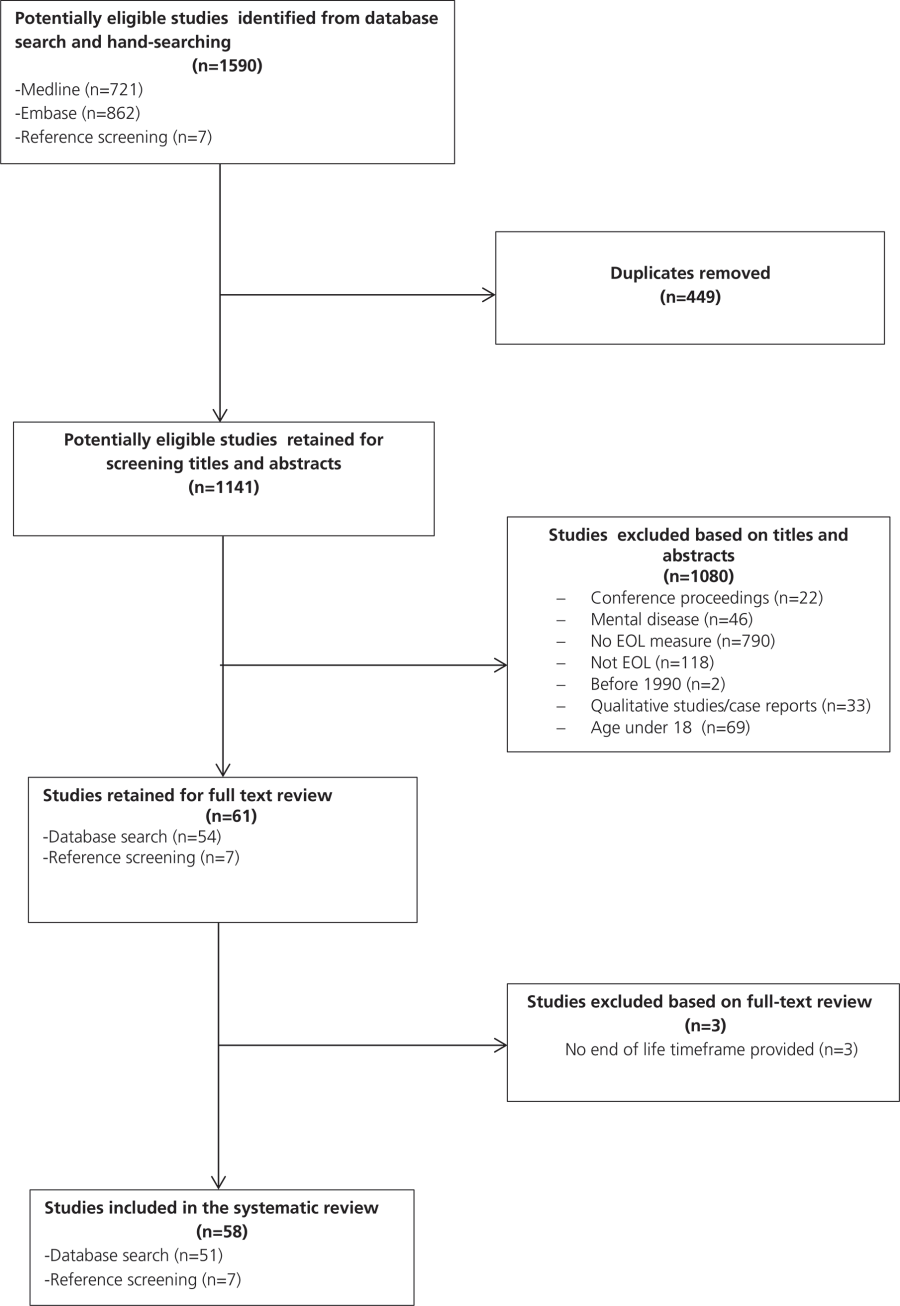
Identification of eligible articles on measures of intensity of end of life care.

We identified 1590 potentially eligible studies and included 58 studies that met our inclusion criteria and described measures of intensity of end of life care in the last year of life (Fig 1).

### Characteristics of studies

Characteristics of included studies are provided in Table 1. The majority of studies were in populations aged 65 years and older. Only four studies were gender-specific (i.e. males alone). Three were prospective cohort studies, 53 retrospective cohort studies and two were randomized controlled trials. Almost all studies relied on administrative data. Study aims were heterogeneous. Twenty-four studies looked explicitly at the measures of intensity of care. The remaining studies did not have intensity of care as a primary aim but met our definition (Table 1). They evaluated health care utilization/care patterns at the end of life (n = 17), variation of end of life care across different settings, populations and time trends (n = 14), or evaluated quality of end of life care (n = 3). Studies measured intensity of life care over different timeframes ranging from hours to months, in disease-specific populations (including cancer, heart failure, other chronic disease [e.g., respiratory diseases, end stage renal disease], trauma, multiple diseases [e.g., hip fracture, COPD, colorectal cancer and acute myocardial infraction]) and non-disease-specific populations (Table 1).

**Table 1:** Characteristics of included studies.

Aim	All studies(%) (n = 58)
Intensity of care	24 (41.4)
Health care utilization	17 (29.3)
Time trends in end of life care	14 (24.1)
Quality of end of life care	3 (5.2)
**Year**	
1990–1994	2 (3.4)
1995–1999	1 (1.7)
2000–2004	4 (6.9)
2005–2009	21 (36.2)
2010–2014	30 (51.7)
**Country**	
Asia	4 (6.9)
Australia	1 (1.7)
Europe	8 (13.8)
Northern America	45 (77.6)
**Study design**	
Prospective cohort study	3 (5.2)
Retrospective cohort study	53 (91.3)
Randomised controlled trials	2 (3.4)
**Validity**	
Criterion validity	3 (5.2)
Content and criterion validity	1 (1.7)
**Summary score**	
Included^a^	2 (3.4)
**Minimum age for inclusion (years)**	
18+	4 (6.9)
60+	30 (51.7)
Other^b^	24 (41.4)
**Disease**	
Cancer	31 (53.4)
Chronic diseases^c^	5 (8.6)
Heart failure	2 (3.4)
Trauma	1 (1.7)
Multiple diseases^d^	3 (5.2)
Non-disease specific	16 (27.6)
**End of life timeframe**	
≤1 month	23 (39.7)
2 months	1 (1.7)
3 months	1 (1.7)
6 months	18 (31.0)
12 months	15 (25.9)
**Number of measures reported in each study**	
Single measure^e^	7 (12.0)
More than one measure (range 2–46)	51 (87.9)
**Quality of evidence**	
Good	9 (15.5)
Moderate	46 (79.3)
Low	3 (5.1)

^a^One study included six individual measures (chemotherapy use, >1 ED visit, >1 hospital admission, >14 days in hospital, >1 ICU admission, or death in hospital), and one study included seven individual measures (ICU admission, ICU LOS, intubation and mechanical ventilation, haemodialysis, tracheostomy, gastrostomy).

^b^Reported age as mean and median.

^c^Chronic diseases (e.g., respiratory diseases, end—stage renal disease).

^d^Hip fracture, COPD, colorectal cancer and acute myocardial infraction.

^e^Included only one measure (e.g., radiotherapy use, chemotherapy use).

### Types of measures

We organized measures into three key domains of end of life care: hospitalizations, life-sustaining invasive procedures and life-prolonging treatments. The majority of studies used more than one measure. Table 2 provides an overview of the measures by domain with descriptions of each measure.

**Table 2:** Summary of intensity of end of life care measures from included studies.

Category of care	Description of measures	N	References
			** **
	No. of hospitalizations(count, mean, median, %, rate, SD, categorical yes/no)	44	[11, 12, 23, 24, 40–79]
	Number of hospital re-admissions (count)		
	Admitted to the hospital (yes/no)		
	Hospital LOS (days, months, median, categorical cut—off e.g.>1 >2, >14 days)		
	ICU admission (count, median, [IQR], rate, %, SD, yes/no)	39	[11–13, 21–24, 41, 42, 44, 45, 47, 52–59, 65–68, 72–86]
**Hospitalizations**	Any ICU admission (count)		
	ICU LOS (days, mean, median, categorical yes/no, categorical cut-off e.g., 0, 1, ≥2)		
	ICU admission during hospital stay (yes/no)		
	Terminal ICU admission (rate, %)		
	ED visits (mean, %, rate, categorical yes/no, categorical cut off of ED visits)	24	[24, 40, 41, 47–51, 53–55, 57, 65–67, 71, 74–76, 79, 80, 84, 85, 87]
	ED with and without hospital admission (categorical yes/no)		
			
			
	Intubation/mechanical ventilation (categorical yes/no, rate, %)	17	[13, 21–23, 45, 51, 52, 56, 58, 66, 68, 72, 77, 82, 83, 86, 87]
	Ventilator days (count of days)		
**Life-sustaining procedures**	CPR (median, mean, categorical yes/no, %)	9	[21, 23, 45, 48, 51, 58, 66, 68, 72]
	Feeding tube placement (rate, categorical yes/no, %)	10	[13, 21, 23, 45, 51, 58, 68, 72, 82, 83]
	Tracheostomy (rate, categorical yes/no, %)	6	[21, 22, 45, 72, 82, 83]
	Dialysis (rate, categorical yes/no, %)	10	[21, 22, 51, 72, 78, 82, 83, 86–88]
	Chemotherapy (count, categorical yes/no, %)	27	[24, 43, 47–51, 53–55, 57, 58, 62, 65–68, 72–76, 80, 84, 85, 87, 89]
	Intravenous chemotherapy (median, range, mean, SD)		
	Oral chemotherapy (median, range, mean, SD)		
	Average number of chemotherapy cycles* (%)		
	Average number of chemotherapy regimens* (%)		
**Life-prolonging treatments**	Blood transfusion (%, categorical yes/no)	2	[72, 87]
	Receipt of radiotherapy (categorical yes/no, %)	10	[41, 43, 48, 49, 51, 53, 76, 80, 87, 90]
	Radiotherapy days (count of days)		
	Underwent medical imaging (categorical yes/no)	5	[13, 48, 56, 72, 87]
	Surgical interventions (diagnostic, curative, elective, palliative (yes/no)	8	[11, 13, 41, 43, 45, 46, 49, 51]
	Surgical admissions (mean, yes/no, %)		

Abbreviations: SD, standard deviation; IQR, interquartile range; ED, emergency department; CPR, cardiopulmonary resuscitation; ICU, intensive care unit; LOS, length of stay.

*Chemotherapy regimens include treatment plans (e.g. type of drugs, instructions on when the drug should be taken) whereas chemotherapy cycles include the time of chemotherapy treatment and the break before the next chemotherapy.

#### Hospitalizations

Measures focusing on hospitalizations were most widely reported (n = 44). They were used most commonly in cancer patients (n = 22), with fewer studies in groups with other illnesses. Intensity of end of life care was reported as number of hospitalizations (count, mean, median, percentage, standard deviation [SD], categorical yes/no), number of re-admissions (count), and hospital length of stay (LOS: count of days, months, median, categorical cut-off e.g. >1, >2, >14 days).

We identified 39 studies that measured intensity of ICU use in the last months of life, as ICU admissions (count, median interquartile range [IQR], rate, %, SD) or ICU LOS (count of days, median, categorical cut-off e.g. 0, 1, ≥2 days). Emergency department (ED) admissions were reported in 24 studies, as ED visits (mean, percentage, rate) or ED admissions (with and without hospital admission) and cut-offs of ED visits in the last month of life (0, 1, 2, ≥3).

#### Potentially life-sustaining invasive procedures

Compared with hospitalizations, studies measuring intensity of life-sustaining treatments were less numerous. The most commonly reported life-sustaining treatments were intubation/mechanical ventilation (n = 17), measured as receipt of intubation/mechanical ventilation (yes/no). Other measures included feeding tube placement (n = 10), dialysis (n = 10), CPR (n = 9), and tracheostomy (n = 6). These measures were applied in both disease and non-disease specific populations, but were more widely used in cancer patients.

#### Potentially life-prolonging treatments

Chemotherapy was the most frequently reported life-prolonging treatment (n = 27), described as chemotherapy use (mean, median, range, standard deviation [SD]), average number of cycles and regimens within the last 3–6 months, or last 7–30 days for a range of cancer types (e.g., prostate, lung, breast, colorectal, gastrointestinal, colorectal). Eight studies evaluated intensity of surgical procedures at the end of life (e.g. general, gynaecologic, orthopaedic, thoracic, and urologic or neurosurgical interventions). One study developed a surgical intensity score defined as the proportion of decedents who received a surgical procedure during the last year of life (e.g., any surgery that involved incision, excision, manipulation, suturing of tissue). Ten studies measured receipt of radiotherapy in the last 14–30 days of life. Other less frequently reported measures were number of blood transfusions, and medical imaging.

The vast majority of studies measuring intensity of life-prolonging treatments reported results using more than one measure (e.g. ICU, ED, and chemotherapy).

#### Summary score

Two studies reported results based on an intensity of end of life care summary scores (Table 1). Earle *et al*., (2005) combined six measures (use of chemotherapy, >1 ED visit, >1 hospital admission, >14 days in hospital, >1 ICU admission, or death in hospital) with higher scores (range 0–6) indicating more intensive end of life care. This score was adopted in other studies (Tang et al., 2009). Lin et al., (2009) combined seven measures (ICU admission, ICU LOS, intubation and mechanical ventilation, haemodialysis, tracheostomy, gastrostomy) also with higher scores (range from -2.08 to 3.12) indicating more intensive end of care.

#### Validity

Four studies involved a panel of experts to assess content validity (n = 3) or criterion validity (n = 1). One study reported both on content and criterion validity. No study reported on construct validity. Earle et al., (2008) explored validity of their intensity of end of life care measures by correlating them with measures of family members’ satisfaction with quality of care near the end of life. The results showed (statistically non-significant) trends toward less satisfaction with care when chemotherapy was administered two weeks before death, death occurred in an acute care setting, or there was no or only a short (≤3 day) hospice stay. Sato and colleagues (2008) assessed content validity and inter-rater reliability using a team of physicians, palliative care doctors and research nurses. Earle and colleagues (2005) used an expert panel of health providers to assess the validity of measures such as chemotherapy, ED/ICU admission, hospitalization, hospice use. Barnato *et al*., (2009) assessed criterion validity by comparing Pennsylvania Health Care Cost Containment Council discharge data (PHC4) intensity of end of life care measures with measures used in the Dartmouth Atlas of Health Care. The study reported correlations for six measures of the PHC4 (ICU admission, ICU LOS, intubation and mechanical ventilation, tracheostomy, gastrostomy tube placement, haemodialysis) and the four Dartmouth Atlas measures (inpatient reimbursement, hospital days, ICU days, medical doctor visits) in two populations (decedents and patients with a high probability of dying on hospital admissions).

### Quality of evidence

We considered the quality of evidence to be good for 9 (15. 5%) studies, moderate for 46 (79.3%) studies and low for 3 (5.1%) studies (Table 1). The most common reason for downgrading the quality of evidence was the lack of validity of measures. There were few studies which we rated as good because the measures were repeatedly adopted in other studies. This was particularly the case with measures developed by Earle *et al*., (2004) and Earle *et al*., (2005).

## Discussion

### Summary of main findings

To the best of our knowledge, this is the first systematic review of measures used for evaluating intensity of end of life care. We assessed studies measuring hospitalizations, life-sustaining invasive procedures and life-prolonging treatments in the last 12 months of life. Aims, populations studied, definitions of intensity of end of life care and measures used were heterogeneous across studies. The number of intensity of end of life care studies grew over the last decade with over 50% of the studies included published within the last five years. Our findings show that intensity of end of life care is most commonly evaluated using a combination of measures, including two summary score measures. This review also highlights an important deficit in health services research of end of life care. Measures of intensity of end of life care, although widely used in health services research, lack validation and general agreement by experts in the field. Overall, we consider the evidence to be of moderate quality. It could be argued that the lack of prospective studies reflects the difficulty of conducting research with people who are approaching death [29].

### Strengths and weaknesses of the systematic review

This systematic review was based on a comprehensive literature search that resulted in the inclusion of 58 studies that reported on a variety of intensity of end of life care measures. We collected an extensive list of details on the studies and the measures that researchers used. The greatest strength of this systematic review is that it provides a unique and detail-rich overview of measures of intensity of end of life care used in a wide variety of settings.

There is no agreement on the end of life timeframe, which ranges in the literature from hours to months [16]. The World Health Organization [30] has a published a commonly used definition for palliative care, but it includes neither timeframe, nor terminology for intensity of care, nor a definition of end of life care. Previous research also revealed no general agreement on the best interval or measures for identifying end of life care [31]. As a result, we may have missed some potentially relevant studies on end of life care because they did not fall within our strict definition. We also focused only on services, and did not include other settings like outpatient clinics, hospice or home care. We included all studies that met our definition but were unable to account for differences in definition. We recognize that our definition may not be entirely consistent with other definitions of intensity of end of life care. We do not suggest that ours is the best definition but regard it as good working definition that represents a broad set of health services research designed to evaluate the intensity of end of life care.

### Challenges in measuring the intensity of end of life care

Our findings should be interpreted carefully, since many of the studies we included did not focus on measuring intensity of end of life care alone. Less than half of the studies we reviewed were based on an explicit definition or primarily aimed to study intensity of end of life care. Many measures included in this review were used to evaluate health care utilization more generally. However, the measures often overlapped despite differences in objectives. Thus it remains unclear if measures developed specifically to assess intensity of care are necessary. Health services utilization measures answer questions about volume of care [32] while measures of intensity of care tend to be more disease specific. For example, measures of aggressiveness of care (e.g. frequent ED visits or hospitalizations, long inpatient LOS) are nearly exclusively used in cancer populations to assess poor quality of end of life care [24]. The paucity of validation studies makes distinguishing between measures and their specific uses difficult. These two sets of measures originate from differences in the purposes of studies (e.g., a health service perspective vs. a clinical perspective). Both sets of measures are potentially useful and a better understanding of which measures should be used in which settings would be instrumental to guiding health service research in the future.

Many of the examined studies actually repeatedly utilize a single measure set, those measures developed by Earle, C.C., et al. (2004). These measures have subsequently been adopted for various uses, including by the American Society of Clinical Oncology's QOPI (quality oncology practice initiative) measure set (http://www.asco.org/). The repeated use of this measure set reflects the influence of these measures, whether or not they have been formally validated.

Our study shows that hospitalization rates at the end of life are high, regardless of the specifications of the measure selected. High number of hospitalizations at the end of life may be related to lack of structure and availability of homecare services. Previous studies suggest that increased end of life homecare services will reduce the demand for acute care services at the end of life as well as improve the quality of life of these patients [24, 33, 34]. Furthermore, treatment delivered at the end of life may also be related to the region of care. [1, 35]. Unfortunately, given the mass of papers from North America and small numbers from other regions it was not possible to adequately examine results by region. There was a different pattern of care for cancer patients than for the non-disease specific group. Measures developed for cancer patients are well documented and over represented in the literature. However, the majority of these studies reuses data primarily collected for administrative purposes thus restricting any potential influence to a non-measurable unsystematic bias.

These measures generally examined the last month of life, when cancer patients are most likely to be hospitalized. The trajectory to death is easier to identify for cancer patients than the trajectory for patients with other diseases, and this may account for the difference. Measures designed for cancer care may not be appropriate for other disease, and more research on end of life measures should be conducted on populations with other diseases like heart failure. However, measures developed for general populations may not be specific enough to identify areas for quality improvement. Measures also vary between countries, perhaps due to the wide range of health policies, and organizational structures, across countries.

Most research on intensity of end of life care is based on retrospective cohort studies that use administrative data because it is only possible to determine the exact period before death retrospectively [15, 20]. Thus, the most readily available sources of healthcare use are administrative datasets. Most studies retrospectively assessed the care received by patients in the time frame before death, but one study identified patients who were entering the terminal phase of disease, and whose probability of death was high, and then prospectively observed patient care forward in time [21, 36]. Each approach has advantages and disadvantages. Measures based on treatments given to patients with a high probability of dying may accurately identify end of life patients and be less prone to bias [37, 38]. Researchers argue that, in order for the results to be valid, the care of end of life patients must be captured prospectively [19]. But the higher quality of retrospective data may produce results more useful for monitoring end of life care across providers, geographic areas, demographic groups, and time periods [37].

An analysis of the qualitative literature on the intensity of end of life care was beyond the scope of this study. Several qualitative tools have been developed to measure different aspects of end of life, including quality of life, physical symptom control, emotional and cognitive symptoms, spirituality, grief and bereavement, satisfaction and quality of care, and caregiver well-being [39].

## Conclusions

There is no consensus on the definition for intensity of end of life care. The associated measures are seldom validated and often used for varying aims, in differing populations and most commonly in combinations of more than one at a time. Definitions, methods, and strategies all vary across studies and countries. The choice and assessment of measures of intensity of care at the end of life should be based on the aim of the study although which measure suits specific aims best remains unclear. This review is the first to attempt to identify measures used specifically for evaluating intensity of end of life care. It provides a synthesis for choosing measures based on their previous use but also highlights the crucial need for more validation studies.

## Supporting Information

S1 Checklist PRISMA checklist. (PDF)

S1 Search Strategy Search strategy for Ovid MEDLINE. (PDF)

S1 Table Summary table of all included studies. (PDF)

S2 Table Summary table of excluded studies. (PDF)
