# Supplementary material for: Measuring Intensity of End of Life Care: A Systematic Review
Source: PLoS One. 2015 Apr 14;10(4):e0123764. doi: 10.1371/journal.pone.0123764 (PMC4396980; doi:10.1371/journal.pone.0123764)
Supplement: S1 Search Strategy — (PDF) [file pone.0123764.s002.pdf]

Search strategy for Ovid MEDLINE

1. End of life.ab,ti,tw.
2. End of life care.ab,sh,ti,tw.
3. EOL care.ab,ti,tw.
4. EOLC.ab,sh,ti,tw.
5. Dying.ti,sh,ab,tw.
6. At life's end.ab,ti,tw.
7. (End adj5 life).ab,ti,tw.
8. (Last adj5 life).ab,ti,tw.
9. (Final adj5 life).ab,ti,tw.
10. (Last adj5 dying).ab,ti,tw.
11. (Final adj5 dying).ab,ti,tw.
12. (Last adj5 death).ab,ti,tw.
13. (Final adj5 death).ab,ti,tw.
14. Fatal disease*.ab,ti,tw.
15. Fatal illness*.ab,ti,tw.
16. Terminal.ab,ti,tw.
17. Critically ill*.ab,ti,tw.
18. Terminal care.ab,sh,ti,tw.
19. Fatally ill*.ti,ab,tw.
20. (Terminal adj3 patient*).ab,ti,tw.
21. "Terminal illness*".ab,ti,tw.
22. "Terminal disease*".ab,ti,tw.
23. "Terminal phase*".ab,ti,tw.
24. "Terminal sickness*".ab,ti,tw.
25. "Terminal stage*".ab,ti,tw.
26. "Terminal hospitali?ation*".ab,ti,tw.
27. (Terminally adj3 patient*).ab,ti,tw.
28. Seriously ill.ab,ti,tw.
29. Severely ill.ab,ti,tw.

30. Palliative care.ab,ti,tw.

31. "Palliative therapy*".ab,ti,tw.

32. "Palliative stage*".ab,ti,tw.

33. "Palliative phase*".ab,ti,tw.

34. (Palliative adj3 patient*).ti,ab,tw.

35. Hospice care.ab,ti,tw.

36. Treatment intensity.ab,ti,tw.

37. "Treatment activity*".ab,ti,tw.

38. (Intensity adj3 treatment).ab,ti,tw.

39. Surgical intensity.ab,ti,tw.

40. Treatment frequency.ab,ti,tw.

41. Care frequency.ab,ti,tw.

42. Treatment severity.ab,ti,tw.

43. Care severity.ab,ti,tw.

44. "Pattern* of treatment".ab,ti,tw.

45. "Pattern* of care".ab,ti,tw.

46. (patterns adj5 "use").ab,ti,tw.

47. "Treatment pattern*".ab,ti,tw.

48. "Care pattern*".ab,ti,tw.

49. Care intensity.ab,ti,tw.

50. (Intensity adj3 care).ab,ti,tw.

51. (Intensity adj3 treatment).ab,ti,tw.

52. (Frequency adj3 treatment).ab,ti,tw.

53. Care consumption.ab,ti,tw.

54. (frequency adj8 care).ab,ti,tw.

55. (consumption adj3 care).ab,ti,tw.

56. (Aggressiveness adj3 care).ab,ti,tw.

57. (Aggressiveness adj3 treatment).ab,ti,tw.

58. (Quantity adj3 care).ab,ti,tw.

59. (Amount adj3 care).ab,ti,tw.

60. Care utili?ation.ab,ti,tw.

61. "Health services utili?ation*".ab,ti,tw.

62. "use of hospital*".ab,ti,tw.

63. 1 or 2 or 3 or 4 or 5 or 6 or 7 or 8 or 9 or 10 or 11 or 12 or 13 or 14 or 15 or 16 or 17 or 18 or 19 or 20 or 21 or 22 or 23 or 24 or 25 or 26 or 27 or 28 or 29 or 30 or 31 or 32 or 33 or 34 or 35

64. 36 or 37 or 38 or 39 or 40 or 41 or 42 or 43 or 44 or 45 or 46 or 47 or 48 or 49 or 50 or 51 or 52 or 53 or 54 or 55 or 56 or 57 or 58 or 59 or 60 or 61 or 62

65. 63 and 64

66. limit 65 to humans
