# Supplementary material for: Measuring Intensity of End of Life Care: A Systematic Review
Source: PLoS One. 2015 Apr 14;10(4):e0123764. doi: 10.1371/journal.pone.0123764 (PMC4396980; doi:10.1371/journal.pone.0123764)
Supplement: S1 Table — (PDF) [file pone.0123764.s003.pdf]

Supplementary table S1. Summary table of all included studies

Author, year, country	Aim of the study	Study design	Disease	Age/Gender	Measure	End of life timeframe	Validity	Summary score	Quality of evidence
Alonso-Babarro, A., et al. (2013), Spain	To examine the frequency of hospital deaths and the use of hospital among cancer patients	Retrospective cohort study	Cancer	18+<br>All	Visited ER (count, mean, yes/no)<br>Admitted to the hospital (count, mean, yes/no)	Last 2 months	Not reported	Not reported	Moderate
Alturki, A., et al. (2014), Canada	To determine the variation in care among persons dying of primary intracranial tumors (ICT)	Retrospective cohort study	Cancer	18+<br>All	ICU admissions (0, 1, ≥2)<br>ER visits (0, 1, ≥3)<br>Time spent in hospital (0-6 months)<br>Duration of last admission leading to death in acute care (>7 days, ≥7 days)<br>Received neurosurgical intervention (0, ≥1)<br>Received radiotherapy (yes/no)	Last 6 months	Not reported	Not reported	Moderate
Au, D. H., et al. (2006), United States	To examine care use and geographical variation among patients with COPD compared lung cancer	Retrospective cohort study	COPD and lung cancer	70.6 (mean)<br>Not reported	Hospital admission (count, %)<br>Hospital days (median, [IQR])<br>ICU utilization (count, %)<br>ICU LOS (days, median, IQR)	Last 6 month	Not reported	Not reported	Moderate
Axelsson, B.(1997), Sweden	To examine medical utilization of incurable cancer patients	Retrospective cohort study	Cancer	76 (median)<br>All	Terminal hospitalization (median)<br>Hospital LOS (days)<br>Received surgery, radiotherapy and chemotherapy (yes/no, count, %)	Last 6 months	Not reported	Not reported	Low
Barbera, L., et al. (2008), Canada	To describe the aggressiveness of care in lung cancer patients and differences between lung cancer patients who died in an acute care hospital and a lung cancer patients who were admitted to hospital but died elsewhere	Retrospective cohort study	Lung cancer	20+<br>All	At least one visit to the emergency room (%)<br>Admitted to the intensive care unit (%,)<br>Received a chemotherapy injection (%)<br>Chemotherapy during hospital admission (%)<br>Radiotherapy during admission (%,)<br>ICU during admission (%)	Last 2 weeks	Not reported	Not reported	Moderate

Barnato, A. E., et al. (2006), United States	To determine if racial and ethnic variations exist in intensive care (ICU) use during terminal hospitalizations and determine if they they can be explained by systematic differences in hospital utilization by race/ethnicity	Retrospective cohort study	Non-disease specific	18+<br>All	Terminal ICU admission (rate, %)<br>Use of ICU services (%)<br>ICU LOS (days)	Last 12 months	Not reported	Not reported	Moderate
Barnato, A. E., et al. (2010), United States	To explore the relationship between hospital EOL treatment intensity and post admission survival	Retrospective cohort study	Non-disease specific	65+<br>All	ICU admission (rate)<br>ICU LOS<br>Intubation/mechanical ventilation (rate)<br>Tracheostomy (rate)<br>Hemodialysis (rate)<br>Gastrostomy (rate)	Last 6 months	Not reported	Not reported	Moderate
Barnato, A. E., et al. (2007), United States	To examine intensive care unit and intensive procedure use by race, using utilization among survivors for comparison	Retrospective cohort study	Non-disease specific	65+<br>All	One or more ICU admission (%)<br>ICU admission (%)<br>Intubation and tracheostomy (%)<br>Feeding tube placement (%)<br>Hemodialysis (%)	Last 12 months of life	Not reported	Not reported	Moderate
Barnato, A. E., et al. (2009), United States	To develop and validate new measures of hospital end of life treatment intensity	Retrospective cohort study	Hospital<br>Non disease specific	65+<br>All	ICU admissions (median, mean SD)<br>ICU LOS (days)<br>Intubation/mechanical ventilation (median, mean SD)<br>Tracheostomy (median, mean SD)<br>Gastrostomy tube (median, mean SD)<br>Hemodialysis (median, mean SD)<br>CPR (median, mean SD)	Last 6 months	Construct validity was assessed by comparing a new EOL intensity of care measure (the PHC4) with an Dartmouth Atlas measures	Not reported	Good
Barnato, A. E., et al. (2007), United States	To test whether variations in (EOL) treatment intensity are associated with regional differences in patient	Cross-sectional survey	Hospital<br>Non-disease specific	65+<br>All	Hospital LOS (days)<br>ICU LOS (days)	Last 6 months	Not reported	Not reported	Good

	preferences for EOL care								
Barnato, A. E., et al. (2004), United States	To determine whether increases in hospital treatment intensity over time among decedents are responsible for the growth in EOL expenditures	Retrospective cohort study	Non-disease specific	65+<br>All	One or more acute care hospitalizations (%)<br>Average LOS (days, mean)<br>Any ICU admission (%)<br>ICU LOS (days)<br>Intubation and tracheostomy (%, mean)<br>Feeding tube placement (%, mean)<br>CPR (%, mean)<br>Surgical procedures	Last 12 months	Not reported	Not reported	Moderate
Barnet, C. S., et al. (2013), United States	To examine the types procedures that patients undergo in their last year of life	Retrospective cohort study	Non-disease specific	59 (mean)<br>All	Hospital admissions (≥4)<br>LOS (median number of days, [IQR])<br>Underwent gynecologic, general, orthopedic, thoracic, urologic procedures (count, %)	Last 12 months	Not reported	Not reported	Moderate
Bergman, J., et al. (2010), United States	To measure quality of EOLC in low-income uninsured men dying of prostate cancer	Retrospective cohort study	Cancer	66.4 (mean)<br>Males	Chemotherapy (yes/no)<br>New chemotherapy regimen initiated within 3 months of death (count, %)<br>Chemotherapy within 2 weeks of death (count, %)<br>No.of ER visits in last 12 months of life (mean, SD)<br>No. of inpatient admissions in last 3 m of life (mean, SD)<br>No. of intensive care unit stays in last 3 months of life (mean, SD)	Last 12 months	Not reported	Not reported	Moderate
Bergman, J., et al. (2011), United States	To characterize hospice use by men dying of prostate cancer and to compare the use of high-intensity care between those who did or did not enroll in hospice	Retrospective cohort study	Cancer	66+<br>Males	Imaging, radiation (count)<br>CPR (count)<br>Chemotherapy (count)<br>Inpatient admission (count)<br>Emergency department visits (count)<br>>1 Emergency department visit<br>Intensive care unit admissions (count)	Last 6 months	Not reported	Not reported	Moderate
Braga, S., et al. (2007), Portugal	To evaluate chemotherapy among adult patients with advanced solid tumors	Retrospective cohort study	Cancer	61 (median)<br>All	Emergency room visits (count,%, median)<br>Hospital admissions (count, %, median)<br>Chemotherapy (%, median)<br>Surgery (%)	Last 3 months	Not reported	Not reported	Low

					Radiotherapy (median, %)				
Bukki, J. et al. (2013), Germany	To assess determinants of symptom burden and treatment intensity at the end of life	Retrospective chart review	Hematologic cancers solid tumors, unknown conditions	62.4 (mean)	X-ray examination (%)<br>Blood transfusion (%)<br>Emergency hospital admission (%)<br>Chemotherapy (%)<br>Radiation therapy (%)<br>Hemodialysis (%)<br>Mechanical ventilation (%)	Last 3 - 14 days	Not reported	Not reported	Moderate
Chan, K. (2012), Hong Kong	To describe the aggressiveness of EOL in an oncology center in Hong Kong	Retrospective cohort study	Cancer	70 (median)<br>All	Administration of chemotherapy (yes/no)<br>New chemotherapy regimen within 30 days of death (yes/no)<br>Receiving chemotherapy within 14 days of death (yes/no)<br>More than 1 ER visit in the last month of life (yes/no)<br>Admission to acute hospitals (yes/no)	Last 14-30 days	Not reported	Not reported	Moderate
Cintron, A., et al.(2003) United States	To describe hospitalization of hospice patients with cancer	Retrospective cohort study	Cancer	66+<br>All	Hospital length of stay in days (median)<br>Hospital admission to death in days (median)<br>Major surgery (count, %)<br>Minor surgery (count, %)<br>Chemotherapy (count, %)<br>Radiotherapy (count, %)<br>Intubation/ventilation (count, %)<br>CPR (count, %)<br>Dialysis (count, %)<br>Feeding tube placement (count, %)	Last 12 months	No reported	Not reported	Moderate
Cooper, Z., et al. (2012), United States	To determine if racial disparities exists in intensity of EOL of trauma patients	Prospective cohort study	Trauma patients	18 – 84<br>All	ICU admission<br>Mechanical ventilation (ventilation days)<br>Length of stay (days)	Last 12 months	Not reported	Not reported	Moderate
Cowall, D. E., et al. (2012), United States	To evaluate data with cancer community in order to determine EOL cancer practices	Retrospective cohort study	Cancer	70 (mean)<br>All	Chemotherapy in last 14 days<br>Chemotherapy in last 30 days<br>More than one emergency room visits in last 30 days<br>Admission to ICU in last 30 days (yes/no)<br>More than one hospitalization (yes/no)<br>Received radiotherapy in the last	Last 14- 30 days	Not reported	Not reported	Moderate

					14 days of life (yes/ no)<br>Started radiotherapy in last 30 days				
Earle, C. C., et al. (2008), United States	To review the literature on aggressiveness of cancer care near the end of life	Retrospective cohort study	Cancer	65+<br>All	More than one ER visit in the last month of life (%)<br>Last dose of chemotherapy within 14 days of death (%)<br>ICU admission in the last month of life (%)<br>More than one hospitalization in the last month of life (%)<br>Last chemotherapy regimen started within 30 days of death (%)	Last 14-30 days	Content validity conducted a survey that contained 20 questions about satisfaction with symptom control, psychosocial care, information provision, and availability of providers	Not reported	Good
Earle, C. C., et al. (2004), United States	To characterize the aggressiveness of end-of-life cancer treatment for older adults on Medicare, and its relationship to the availability of healthcare resources	Retrospective cohort study	Cancer	65+<br>All	Proportion starting a new chemotherapy regimen within 30 days of death<br>Proportion receiving chemotherapy within 14 days of death<br>Proportion with > 1 ER visit in the last month of life<br>Proportion with >14 days in hospital in the last month of life<br>Proportion with > 1 hospital admission in the last month of life<br>Proportion admitted to the ICU in the last month of life	Last 14-30 days	Not reported	Not reported	Good
Earle, C. C., et al. (2005), United States	To evaluate measures that could use existing administrative data to assess the intensity of end-of-life cancer care	Retrospective cohort study	Cancer	65 +<br>All	Proportion receiving chemotherapy in the last 14 days of life<br>Proportion receiving chemotherapy in the last 30 days of life<br>More than one ER visit in the last month of life<br>More than one hospitalization in the last month of life<br>Admission to the ICU in the last month of life	Last 14 - 30 days	Content validity was checked by and an expert panel of health providers that identified items to measure end-of-life intensity of care (chemotherapy, ER, hospitalization,	The authors used six measures (use of chemotherapy, >1 ED visit, >1 hospital admission, >14 days in hospital, >1 ICU admission, or death in	Good

							ICU, hospice in the last days of life)	hospital) with higher scores (range 0-6)	
Fisher, E. S., et al. (2003), United States	To determine whether regions with higher Medicare spending provide better care.	Cohort study	Hip fracture, colorectal cancer, and AMI	65+<br>All	ICU LOS (days)<br>Emergency intubation (yes/no)<br>Feeding tube placement (yes/no)<br>Surgical procedures<br>MRI imaging<br>CT	Last 6 months	Not reported	Not reported	Good
Gielen, B., et al. (2010), Belgium	To examine age variation in place of death, transitions between care settings	Retrospective cohort study	Cancer, non-cancer	41+<br>All	Hospital admission (%)<br>Hospital LOS (days)<br>Admitted into the ICU (%)<br>Received artificial ventilation (%)<br>Medical imaging	Last 6 months	Not reported	Not reported	Moderate
Giovanis, P., et al.(2010), Italy	To analyze aggressiveness of cancer care and consumption of opioids	Retrospective cohort study	Cancer	65 (median)<br>All	Received chemotherapy (%, yes/no)	Last 14-30 days	Not reported	Not reported	Moderate
Gonsalves, W. I., et al. (2011), United States	To examine aggressiveness of care near the EOL among cancer patients before and after PCS	Retrospective cohort study	Cancer	74 (median)<br>All	Chemotherapy (%)<br>More than one emergency department visit (%)<br>Hospitalized for more than 14 days (%)<br>More than one hospital admission (%)<br>ICU admission (%)	Last 14- 30 days of life	Not reported	Not reported	Moderate
Goodman, D.et., al. (2010), United States	To examine quality of end-of-life cancer care for Medicare Beneficiaries	Retrospective cohort study	Cancer	65+<br>All	Percent hospitalized<br>All hospital days per patient<br>Percent admitted to ICU<br>Chemotherapy, intubation, CPR, feeding tube placement	Last 14-30 days, last 6 months	Not reported	Not reported	Good
Goodman, D.et., al.(2011), United States	To investigate trends and variation in EOL care for patients with Severe Chronic Illness	Retrospective cohort study	Chronic diseases	65+<br>All	Hospital days<br>Intensive care days (ICU)	Last 6 months	Not reported	Not reported	Good
Goodridge, D., et al. (2008), Canada	To contrast health care utilization by people with COPD and those with lung cancer in the last 12 months of life	Retrospective cohort study	COPD, lung cancer	77 (median)<br>All	Hospitalization<br>Median average LOS [IQR])<br>% With mean LOS>7 days<br>Median total LOS (IQR)<br>% With any hospitalization<br>% With >2 hospitalizations	Last 12 months	Not reported	Not reported	Moderate

Goodridge, D., et al. (2010), Canada	To examine rural/urban differences in health care utilization and place of death for persons with respiratory illness in the last 12 months of life	Retrospective cohort study	Respiratory disease	77 (mean)<br>All	Hospitalization<br>Median average LOS (IQR)<br>% with mean LOS>7days<br>Median total LOS >7 days<br>% with any<br>% with>2	Last 12 months	Not reported	Not reported	Moderate
Greer, J., et al (2012), United States	To investigate whether early palliative care also affects the frequency and timing of chemotherapy use and hospice care for these patients.	Randomized controlled trial	Cancer	64 (mean)<br>All	Chemotherapy use (count, %)<br>Any within 60 days of death (count, %)<br>Any within 30 days of death (count, %)<br>Any within 14 days of death (count, %)<br>Days between last dose and death for final chemotherapy regimen (count, %)<br>Intravenous (median, range, mean SD)<br>Oral (median, range, mean SD)	Last 14 - 60 days	Not reported	Not reported	Moderate
Henderson, M.,et al. (1990), UK	To determine whether people 65 and over and those who died at spent more of in hospital than those who died younger, and whether the increase in longevity was accompanied by increased time spent in hospital	Retrospective cohort study	Non disease specific	64+<br>Not specified	Total time spent in hospital in last year of life	Last 12 months	Not reported	Not reported	Moderate
Henderson, M.,et al.(1992), UK	To investigate inter-district comparison of hospitalization of elderly people	Retrospective cohort study	Non disease specific	65+<br>Not specified	Total number of days spent in hospital by each person in the final year of life	Last 12 months	Not reported	Not reported	Moderate

Ho, T. H., et al.(2011), Canada	To describe trends in the aggressiveness of EOL cancer care and to compare with findings reported in the United States.	Retrospective cohort study	Cancer	20+<br>Not specified	Last dose of chemotherapy received within 14 days of death<br>More than one emergency department (ED) visit within 30 days of death<br>More than one hospitalization within 30 days of death<br>At least one intensive care unit (ICU) admission within 30 days of death	Last 14-30 days	Not reported	Not reported	Moderate
Keam, B., et al. (2008), Korea	To examine the appropriateness of chemotherapy and care in Korean cancer patients near the end-of-life	Retrospective cohort study	Cancer	59 (mean)<br>All	Proportion receiving chemotherapy within 6 months of deaths (%)<br>Proportion receiving chemotherapy within 2 months of deaths (%)<br>Proportion receiving chemotherapy within 14 days of deaths (%)<br>Average no. of days between last chemotherapy and death days (median, SD)<br>Average no. of regimens (median, SD)<br>Average no. of cycles (median, SD)<br>Average no.of ER visits (median, SD)<br>Average no.of ER visits in the last month of lif (median, SD)<br>Proportion with > 1ER visit in the last month of life (%)<br>Proportion admitted to the ICU in th last month of life (%)	Last 30 days, last 1- 6 months	Not reported	Not reported	Moderate
Keating, N. L.et al., (2010), United States	To compare the aggressiveness of EOL care of older metastatic cancer patients treated in the Veterans Health Administration (VHA) and those under Medicare beneficiaries	Retrospective cohort study	Cancer	66+<br>Males	Aggressive chemotherapy<br>No. and % last dose received within 14 days of death<br>ICU admissions<br>No. and % with at least 1 admission in last month of life<br>Frequent ER visits<br>No. and % with more than ER visit in last month of life	Last 14 -30 days	Not reported	Not reported	Moderate

Kwok, A. C., et al. (2011), United States	To examine national patterns of surgical care in the US among elderly fee-for-service Medicare beneficiaries in their last year of life	Retrospective cohort study	Non-disease specific	65+ All	Surgical intensity<br>The receipt of surgical procedure (incision, excision, manipulation or suturing of tissue) (%)<br>Number of hospital admissions (mean)<br>Number of days in hospital (mean)<br>Number of days in intensive care (mean)	Last 12 months	Not reported	Not reported	Moderate
Lin, C. Y., et al. (2009), United States	To explore the association between organizational factors and EOL treatment intensity in Pennsylvania (PA) hospitals	Retrospective cohort study	Non-disease specific	65+ All	ICU admission (rate)<br>ICU length of stay (days)<br>Intubation and mechanical ventilation (rate)<br>Hemodialysis (rate)<br>Tracheostomy (rate)<br>Gastrostomy (rate)	Last 6 months	Not reported	The summary score includes seven measures (ICU admission, ICU LOS, intubation and mechanical ventilation, hemodialysis, tracheostomy, gastrostomy) Range from -2.08 to 3.12	Moderate
Mack, J. W., et al. (2012), United States	To evaluate the extent to which EOL discussions lead to less aggressive care near death	Prospective cohort study	Cancer	21+ All	Chemotherapy in the last 14 days of life (yes/no)<br>More than one emergency room visit in the last 30 days of life<br>More than one hospitalization in the last 30 days of life<br>More than 14 inpatient hospital days in the last 30 days of life<br>Intensive care unit care in the last 30 days of life (ICU) admission date in the last 30 days of life<br>CPR<br>Ventilator<br>Intubation	Last 14-30 days	Not reported	Not reported	Moderate
Miesfeldt, S., et al. (2012), United States	To measure intensity of end-of-life care for Medicare beneficiaries with	Retrospective cohort study	Cancer	65+ All	More than 1 hospitalization in the last 30 days of life<br>More than one ED visit in the last 30 days of life<br>Admission to ICU in the last 30	Last 14-30 days	Not reported	Not reported	Moderate

	cancer				days<br>Proportion receiving chemotherapy in the last 14 days of life<br>Proportion receiving chemotherapy in the last 30 days of life				
Morden, N. E., et al. (2012), United States	To examine the association between hospital characteristics and eleven end-of-life care measures	Retrospective cohort study	Cancer	66 -99<br>All	Hospitalized, last month of life (%)<br>Hospital LOS (days)<br>ICU use, last month of life (%)<br>ICU LOS (days)<br>Chemotherapy, last 14 days of life (%)<br>Feeding tube (yes/no)<br>CPR(yes/no)<br>Mechanical ventilation (yes/no)	Last 6 months	Not reported	Not reported	Moderate
O'Hare, A. M., et al. (2010), United States	To determine how ESRD treatment practices for older adults vary across regions with differing EOLC	Retrospective cohort study	End-stage renal disease	65+<br>All	Use of peritoneal dialysis (vs hemodialysis) (%)	Last 6 months	Not reported	Not reported	Moderate
Ramroth, H., et al. (2006), Germany	To describe hospitalizations of nursing homes	Retrospective cohort study	Non-disease specific	84.7 (mean)<br>All	Hospitalizations (rates, count, mean)<br>12–4 months before death<br>≤3 months before death	Last 12 months	Not reported	Not reported	Low
Reed, S. D., et al.(2012), United States	To characterize inpatient resource use and costs of patients with heart failure who died of sudden cardiac attack	Randomised controlled trial	Heart failure	61 (mean)<br>All	Hospitalized at least once<br>Number of hospitalizations (mean± SD, median [IQR])<br>Number of inpatient days (mean ± SD, Median [IQR])	Last 12 month	Not reported	Not reported	Moderate
Richardson, S. S., et al. (2007), United States	To analyze whether acute care patients without dementia are more likely to receive aggressive EOLC compared with patients with dementia.	Retrospective cohort study	Patients with and without dementia	67+<br>All	ICU admission (%)<br>Ventilator (%)<br>Dialysis (%)	Last 30 days	Not reported	Not reported	Moderate
Rosenwax, L. K., et al. (2011), Australia	To describe hospital and ED use by people for whom	Retrospective cross-sectional	Cancer, non - cancer	65+<br>All	Number of hospital admissions (mean, SD, median)<br>ER (mean, count)	Last 12 months	Not reported	Not reported	Moderate

	death from cancer or one of another nine conditions was an expected outcome	study			Hospital LOS (days, mean SD)				
Sato, K., et al. (2008), Japan	To assess the quality of end-of-life treatment for dying cancer patients in general wards and palliative care unit	Retrospective chart review	Cancer	21+<br>All	Treatment in the last 48 hours<br>CPR (count, %)<br>Mechanical ventilation (count, %)<br>Tracheostomy (count, %)<br>Intubation (count, %)<br>Dialysis (count, %)<br>Diagnostic testing (radiography, CT scan, count, %)<br>Blood transfusion (count, %)<br>Tube feeding (count, %)<br><br>Proportion starting a new chemotherapy regimen within 30 days of death (count, %)<br><br>Proportion receiving chemotherapy within 14 days of death count, %)<br><br>Proportion admitted to the ICU in the last month of life (count, %)<br><br>Proportion with more than 14 days in hospital in the last month of life (count, %)	Last 48 h, last 14-30 days	Content validity was checked by two palliative care doctors and two research nurses. Three physicians checked the questionnaires. They also checked for inter-rater reliability.	Not reported	Good
Sheffield, K. M., et al. (2011), United States	To characterize hospice use and aggressiveness of care for pancreatic cancer patients	Retrospective cohort study	Cancer	All<br>All	Chemotherapy (%)<br>Acute hospitalization (%)<br>ICU admission (%)	Last 30 days	Not reported	Not reported	Moderate
Smith, A. K., et al. (2009), United States	To examine racial and ethnic variation in use of hospice and high-intensity care in patients with terminal illness	Retrospective cohort study	Cancer	65+<br>All	Two or more hospitalizations (%)<br>More than 14 days in the hospital (%)<br>Intensive care unit (ICU, %)<br>Receipt of chemotherapy in the last 14 days of life (%)<br>Two or more ED visits in the last month of life (%)	Last 30 days	Not reported	Not reported	Moderate

Tang, A. K., et al. (2009), Taiwan	To assess the association between aggressiveness of EOL and patient disease characteristics, primary physician's specialty, hospital characteristics, and availability of health care resources at the hospital and regional levels	Retrospective cohort study	Cancer	65+<br>All	Use of chemotherapy (%)<br>More than one emergency room visit (%)<br>More than one hospital admission (%)<br>More than 14 days of hospitalization (%)<br>Intensive care unit (ICU) admission (mean SD, %)	Last 14-30 days	Not reported	Not reported	Moderate
Temel, J. S., et al. (2008), United States	To describe the aggressiveness of care in newly diagnosed patients with advanced non-small-cell lung cancer	Prospective cohort study	Cancer	66.5 (median)<br>All	Average d between start of last anticancer therapy and death (mean SD, range)<br>Average d between last anticancer therapy dose and death (mean SD, range)<br>New anticancer therapy (chemotherapy, radiation) within 30 d of death (count, %)<br>Any anticancer therapy chemotherapy, radiation within 30 d of death (count, %)<br>Any anticancer therapy chemotherapy, radiation within 14 d of death (count, %)<br>Any anticancer therapy chemotherapy, radiation within 7 d of death (count, %)<br>Emergency department visit within 30 d of death (count, %)<br>>1 emergency department visit within 30 d of death (count, %)<br>Hospital admission within 30 d of death (count, %)<br>>1 hospital admission within 30 d of death (count, %)<br>Average number of inpatient d within 30 d of death (mean SD, range)<br>Admission to the ICU within 30 d of death (n, %)	Last 7-30 days	Not reported	Not reported	Moderate

Teno, J., et al. (2013), United States	To describe changes in site of death, place of care and health care transitions between 2000, 2005, and 2009	Retrospective cohort study	Non-disease specific	66+ All	Hospitalizations in the last 90 days (%) Hospital days last 30- 90 days (mean, median, [IQR]), Three or more hospitalizations in the last 90 days (mean, median, IQR) ICU in the last 30 days (%) ICU days (mean, median, IQR) Mechanical ventilation in the last 30 days (%, yes/no)	Last 30 -90 days	Not reported	Not reported	Moderate
Toole, M., et al. (2012), United States	To examine aggressiveness of cancer care near the end of life	Retrospective cohort study	Cancer	All All	Receiving radiotherapy (yes/no, days on treatment)	Last 30 days	Not reported	Not reported	Moderate
Unroe, K. T., et al.(2011), United States	To examine the resource use in the last 180 days of life	Retrospective cohort study	Heart failure	65+ All	All – cause hospitalizations Inpatient (%) Intensive care unit %) Dialysis (%)	Last 6 months	Not reported	Not reported	Moderate
Warren, J. L., et al. (2011) United States and Ontario	To examine end-of-life care for lung cancer patients in the United States and Ontario	Retrospective cohort study	Cancer	65+ All	ER use (% , rate, mean) ER visits that resulted in hospitalization (%) Hospital use (rate, %, mean) LOS (hospitalization days) ICU (% of hospitalized patients treated in the ICU) ICU LOS (mean length of ICU stay in days)	Last 30 days, last 5 months	Not reported	Not reported	Moderate
Wennberg, J. E., et al. (2004), United States	To evaluate the use of healthcare among patients of US hospitals with strong reputations for high quality care in managing chronic illness	Retrospective cohort study	Chronic illness	65+ All	Number of days spent in hospital (hospital days) Number of days spent in intensive care unit (ICU days)	Last 6 months	Not reported	Not reported	Moderate
Wong, S. P. Y., et al. (2012), United States	To the end-of-life care practices among older Medicare beneficiaries who are receiving long-term dialysis	Retrospective cohort study	End-stage renal disease	65+ All	Hospitalization (%) Hospital LOS (days, mean) Intensive care unit admission (%) Days in an intensive care unit (mean) Mechanical ventilation (%) CPR (%) Feeding tube placement (%)	Last 30 days	Not reported	Not reported	Moderate

***Note:** Measures provided in Table 1 include only those based on our definition. Individual studies may have more measures than listed in Table 1.
