# Supplementary material for: Measuring Intensity of End of Life Care: A Systematic Review
Source: PLoS One. 2015 Apr 14;10(4):e0123764. doi: 10.1371/journal.pone.0123764 (PMC4396980; doi:10.1371/journal.pone.0123764)
Supplement: S2 Table — (PDF) [file pone.0123764.s004.pdf]

Supplementary Table S2. List of full-text excluded articles

**Reason for exclusion:** No clear EOL timeline
1. Angus, D.C., et al., Use of intensive care at the end of life in the United States: an epidemiologic study. Crit Care Med, 2004. 32(3): p. 638-43.<br><br>2. Ohta, B. and J.J. Kronenfeld, Intensity of acute care services at the end of life: nonclinical determinants of treatment variation in an older adult population. J Palliat Med, 2011. 14(6): p. 722-8.<br><br>3. Olsen, M.L., A.L. Bartlett, and T.J. Moynihan, Characterizing care of hospice patients in the hospital setting. J Palliat Med, 2011. 14(2): p. 185-9.
